# Risk factors for skeletal-related events in patients with stage IV lung adenocarcinoma and bone metastases receiving denosumab: a retrospective cohort analysis

**DOI:** 10.1007/s00520-025-10296-0

**Published:** 2026-01-06

**Authors:** Hui Zhang, Daochen Wen, Zhou Liao, Xiaoyong Tan, Hao Wang, Jianxia Xiang, Bing Yan

**Affiliations:** Xuanhan County People’s Hospital, 753 Jiefang Middle Road, East Township, Xuanhan County, Sichuan, China

**Keywords:** Denosumab, Stage IV lung adenocarcinoma, Skeletal-related events, Risk factors, Hypocalcemia, Bisphosphonates

## Abstract

**Purpose:**

This study aimed to identify independent risk factors for skeletal-related events(SREs) during denosumab treatment in patients with bone-metastatic stage IV lung adenocarcinoma by integrating multidimensional clinical parameters, thereby providing an evidence-based foundation for risk stratification and targeted management.

**Methods:**

In this retrospective cohort study, patients with stage IV lung adenocarcinoma and radiologically confirmed bone metastases diagnosed at Xuanhan County People's Hospital between July 2021 and December 2023 were enrolled. All patients received standardized denosumab therapy (120 mg subcutaneously every 4 weeks ± 7 days). Baseline demographic, laboratory, and treatment response data were extracted from the electronic medical record system. Univariate analyses were used to screen candidate variables, followed by multivariate logistic regression to identify independent predictors. Model fit was assessed using the Hosmer–Lemeshow test.

**Results:**

A total of 111 patients were included (median age, 65.5 years; 50.45% male). During follow-up, 27 patients (24.3%) experienced SREs. Multivariate analysis identified a prior history of bisphosphonate use as an independent protective factor against SREs (odds ratio [OR], 0.191; 95% confidence interval [CI], 0.049–0.741 P = 0.017), while Pretreatment hypocalcemia and smoking were both significantly associated with increased risk.

**Conclusions:**

This study is the first to demonstrate in a county-level hospital cohort that a history of bisphosphonate use serves as an independent protective factor against SREs during denosumab treatment, while Pretreatment hypocalcemia and smoking history significantly increase SREs risk. These findings provide critical evidence for individualized management of stage IV lung adenocarcinoma patients with bone metastases.

## Introduction

Lung cancer remains the leading cause of cancer-related mortality worldwide, with its disease burden continuing to rise. According to the Global Cancer Statistics 2022, approximately 2.47 million new cases and 1.76 million deaths from lung cancer were reported globally, accounting for 18.7% of all cancer-related deaths [1]. In China, lung cancer also ranks first in cancer-related mortality, with non–small-cell lung cancer (NSCLC) comprising approximately 85% of all cases [2]. At the time of initial diagnosis, bone metastases are present in approximately 67% of patients with NSCLC, and over half will experience skeletal-related events (SREs) during the disease course [3]. Among NSCLC subtypes, lung adenocarcinoma demonstrates a significantly higher incidence of bone metastases than other histological variants [4]. SREs—which include pathologic fractures, spinal cord compression, bone pain requiring therapeutic intervention, and hypercalcemia—are severe complications that not only substantially impair quality of life but also accelerate disease progression through interactions within the tumor–bone microenvironment, ultimately shortening overall survival [5, 6]. The prevention and mitigation of SREs have thus become central objectives in the comprehensive management of bone-metastatic lung cancer.

Denosumab, a fully human monoclonal antibody targeting receptor activator of nuclear factor-κB ligand (RANKL), inhibits osteoclast-mediated bone resorption by selectively blocking the RANKL–RANK signaling pathway. It is recommended as a first-line bone-targeted agent in patients with solid tumors and bone metastases according to international guidelines [7, 8]. Phase III trials have demonstrated that denosumab significantly delays the time to first SRE and reduces the cumulative risk of subsequent events compared with zoledronic acid in patients with stage IV NSCLC [8, 9]. However, in the subset of patients with stage IV lung adenocarcinoma—a population characterized by a high burden of osseous metastasis and distinct molecular features (e.g., > 50% EGFR mutation rate)—clinical response to denosumab is notably heterogeneous. This observation highlights the need for biomarker-driven stratification strategies to optimize therapeutic decision-making.

Although prior studies have identified several clinical predictors of SREs in patients with NSCLC and bone metastases—including the number of osseous lesions, Eastern Cooperative Oncology Group (ECOG) performance status, smoking history, and hypercalcemia [10–12]—these investigations have notable limitations. First, most existing evidence is derived from heterogeneous NSCLC cohorts without histologic stratification; the distinct molecular characteristics of lung adenocarcinoma, such as high EGFR mutation frequency, may uniquely influence bone microenvironmental dynamics and therapeutic responses, necessitating subtype-specific analyses. Second, few real-world studies have focused specifically on the subset of patients with stage IV lung adenocarcinoma receiving denosumab. To address these gaps, we conducted a retrospective cohort study of 111 patients with bone-metastatic stage IV lung adenocarcinoma treated with denosumab to identify independent predictors of SREs, with a novel focus on the high osseous metastatic burden unique to this clinical population. The findings aim to inform precision bone-targeted therapy in advanced lung adenocarcinoma.

## Methods

### Study design and participants

This single-center retrospective cohort study included consecutive patients with bone-metastatic stage IV lung adenocarcinoma treated with denosumab at Xuanhan County People’s Hospital between July 1, 2021, and December 31, 2023. Eligible patients met the following criteria: (1) age ≥ 18 years; (2) pathologically or cytologically confirmed stage IV lung adenocarcinoma, according to the 8th edition of the American Joint Committee on Cancer (AJCC) staging system; (3) radiologic evidence of bone metastasis or osteolytic lesions (confirmed by CT, MRI, or PET-CT); and (4) no planned orthopedic surgery or palliative radiotherapy to bone. Exclusion criteria included: (1) severe cardiopulmonary dysfunction (New York Heart Association [NYHA] class III–IV heart failure or Global Initiative for Chronic Obstructive Lung Disease [GOLD] stage 4 COPD); and (2) preexisting bone metabolic disorders (such as osteoporosis or Paget’s disease) or a history of prior major skeletal fractures. The study protocol was approved by the Ethics Committee of Xuanhan County People’s Hospital (Approval No. 2022-KSSC-0005). In accordance with the principles of the Declaration of Helsinki, written informed consent was obtained from all enrolled patients prior to the initiation of the study.

### Treatment and follow-up protocol

All patients received systemic therapy according to the 2021 edition of the Chinese Society of Clinical Oncology (CSCO) Guidelines for the Diagnosis and Treatment of Non–Small-Cell Lung Cancer. Denosumab was administered subcutaneously at a dose of 120 mg every 4 weeks (± 7 days). Baseline demographic characteristics, laboratory results, and treatment response data were extracted from the hospital’s electronic medical record system. Treatment continued until the occurrence of intolerable toxicity or disease progression. The follow-up period extended to December 1, 2024. Patients were monitored using a standardized follow-up protocol: (1) medication use and SREs were assessed every 4 weeks; (2) bone metastases were evaluated every 6 months using whole-body bone scintigraphy (99mTc-MDP SPECT/CT) or 18F-FDG PET-CT; and (3) after 12 months of treatment, skeletal evaluation was performed every 3 months using bone CT. Follow-up information was obtained via telephone, text message, outpatient visits, or review of the electronic medical record system.

### Study variables

Key variables of interest included: age, sex, smoking history, Eastern Cooperative Oncology Group performance status (ECOG-PS; ≤ 1 vs. ≥ 2), number of visceral metastatic sites (≤ 1 vs. ≥ 2), type of bone metastasis (osteolytic, osteoblastic, or mixed), genetic mutation status, serum albumin (normal: 35–50 g/L), corrected pretreatment serum calcium (≥ 8.50 mg/dL vs. < 8.50 mg/dL), alkaline phosphatase (normal: 40–150 U/L), lymphocyte count (normal: 1.0–4.8 × 10⁹/L), treatment modality (targeted therapy vs. chemotherapy plus antiangiogenic agents or immune checkpoint inhibitors vs. other therapies), prior bisphosphonate use and its treatment duration. The primary endpoint was the occurrence of skeletal-related events (SREs) during denosumab therapy, defined according to the American Society of Clinical Oncology (ASCO) guidelines as any of the following: (1) pathologic fracture (confirmed independently by two radiologists); (2) spinal cord compression (jointly evaluated by neurologists and orthopedic surgeons); (3) bone pain requiring intervention (numerical pain rating ≥ 4 and treated with radiotherapy); or (4) hypercalcemia (corrected calcium > 11 mg/dL or > 2.75 mmol/L).

### Statistical analysis

All statistical analyses were performed using SPSS version 26.0 (IBM Corp., USA). The Shapiro–Wilk test was used to assess the normality of continuous variables. Normally distributed data were expressed as means ± standard deviation and compared using the independent-samples t test. Non-normally distributed data were reported as medians and interquartile ranges [M (IQR)] and compared using the Mann–Whitney U test. Categorical variables were expressed as counts and percentages [n (%)] and compared using the chi-square test or Fisher’s exact test as appropriate. Variables with P < 0.10 in univariate analyses were entered into a multivariate logistic regression model using forward stepwise selection (entry criterion: likelihood ratio P < 0.05; removal criterion: P ≥ 0.10). Adjusted odds ratios (ORs) and 95% confidence intervals (CIs) were calculated. All hypothesis testing was two-sided, and P < 0.05 was considered statistically significant.

## Results

### Patient selection

**Fig. 1:**
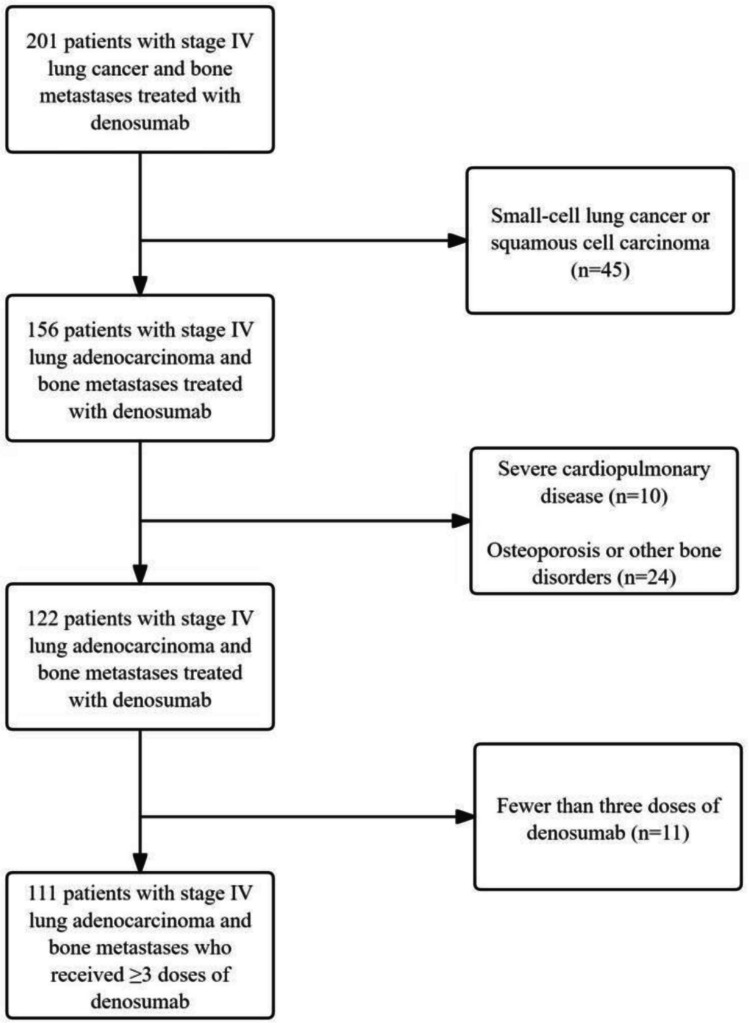
Flow diagram of patient selection and enrollment

Of 201 patients screened, 90 were excluded for the following reasons: small cell or squamous cell carcinoma (n = 45); presence of severe cardiopulmonary comorbidities, osteoporosis, or other bone disorders (n = 34); or fewer than three doses of denosumab (n = 11). A total of 111 eligible patients were included in the final analysis (Fig. 1).

### Baseline characteristics

Baseline characteristics are summarized in Table 1. The median age was 65.5 years (range, 53–73), with 56 men (50.45%) and 55 women (49.55%). The median BMI was 22.63 kg/m^2^ (IQR, 21.22–24.44), and 74 patients (66.67%) had normal body weight (18.5–24 kg/m^2^). A majority (66.67%, n = 74) were never smokers. Most patients (88.29%) had good performance status (ECOG 0 in 31.53%, ECOG 1 in 47.75%). At baseline, 68 patients (61.26%) had already experienced an SRE, Among these patients, 40 presented with isolated bone pain, while 27 had bone pain complicated by either fracture or spinal cord compression. Only one patient presented with isolated fracture without concomitant bone pain. 73 patients (65.77%) had additional sites of metastasis. Osteolytic lesions were the predominant type of bone metastasis (53.15%). A total of 49 patients had confirmed genetic alterations (e.g., EGFR, ALK, or ROS1). Forty-nine patients (44.14%) received targeted therapy, while the remainder received chemotherapy, antiangiogenic therapy, or immunotherapy (monotherapy or in combination). Prior bisphosphonate therapy was documented in 37 patients (33.33%),Among these patients, 33 (89.2%) received zoledronic acid as the primary treatment, while the remaining cases included ibandronate (*n* = 3) and pamidronate (*n* = 1). The median treatment duration was 2.6 months.

**Table 1:** Baseline characteristics and univariate analysis of skeletal-related events (SREs) in stage IV lung adenocarcinoma patients with bone metastases receiving denosumab

Variables		No SRE Group (*n* = 84)	SRE Group (*n* = 27)	*P*
Sex	Male	41(48.81)	15(55.56)	0.659
	Female	43(51.19)	12(44.44)	
Age (Years)		65.5(20.00)	65.50(18.00)	0.652
BMI(kg/m^2^)	< 18.5	5(6.00)	1(3.70)	0.805
	18.5 ~ 24	57(67.90)	17(63.00)	
	≥ 24	22(26.20)	9(33.30)	
Smoking History	No	61(72.60)	13(48.10)	0.033
	Yes	23(27.40)	14(51.90)	
Eastern Cooperative Oncology Group Performance Status (score)	0–1	74(88.10)	24(88.90)	1.000
	2–3	10(11.90)	3(11.10)	
History of Skeletal-Related Events (SREs)	No	32(38.10)	11(40.70)	0.806
	Yes	52(61.90)	16(59.30)	
Number of Visceral Metastatic Sites	≤ 1	55(65.50)	17(63.0)	0.812
	≥ 2	29(34.50)	10(37.00)	
Type of Bone Metastasis	Osteolytic	40(47.60)	19(70.40)	0.100
	Osteoblastic	27(32.10)	6(22.20)	
	Mixed	17(20.20)	2(7.40)	
Genetic Mutation Status	Absent	47(55.95)	15(55.56)	0.971
	Present	37(44.05)	12(44.44)	
Treatment Regimen	Targeted Therapy	37(44.00)	12(44.40)	0.334
	Chemotherapy plus Antiangiogenic Agents/ICIs	26(31.00)	5(18.50)	
	Other Therapies	21(25.00)	10(37.00)	
History of Bisphosphonate Use	Absent	50(59.50)	24(88.90)	0.005
	Present	34(40.50)	3(11.10)	
Duration of bisphosphonate therapy(months)	< 6	53(63.1)	18(66.7)	0.249
	6 ~ 9	23(27.4)	4(14.8)	
	≥ 9	8(9.5)	5(18.5)	
Serum Albumin (g/L)		40.75 ± 4.26	39.83 ± 4.04	0.322
Lymphocyte Count (10⁹/L)		1.08(0.63)	1.22(0.47)	0.392
Alkaline Phosphatase (U/L)		103.5(60.3)	112.0(37.0)	0.201
Corrected pretreatment Serum Calcium (mg/dL)	≥ 8.50	77(91.67)	21(77.78)	0.08
	< 8.50	7(8.33)	6(22.22)	

Exact probability methods were used for variables marked with an asterisk (*); categorical variables were analyzed using the chi-square test. Serum albumin, as a continuous variable with normal distribution, was analyzed using the t test, while other continuous variables were assessed using the Wilcoxon rank-sum test

### Incidence of skeletal-related events

During denosumab treatment, 27 patients (24.32%) experienced SREs. These included 17 cases of bone pain requiring radiotherapy (62.96%), 4 cases of spinal cord compression (14.81%), 3 cases of pathologic fracture (11.11%), and 3 patients who required orthopedic surgery (11.11%).

### Risk factor analysis for SREs

In univariate analysis, smoking history (P = 0.033), prior bisphosphonate use (P = 0.005), and pretreatment hypocalcemia (P = 0.008) were significantly associated with SRE risk (Table 1).

In multivariate logistic regression analysis, prior use of bisphosphonates was independently associated with a reduced risk of SREs (OR, 0.191; 95% confidence interval CI, 0.049–0.741 P = 0.017). In contrast, pretreatment hypocalcemia (OR, 4.913; 95% CI, 1.268 ~ 19.044; P = 0.021) and smoking history (OR, 3.078; 95% CI, 1.131 ~ 8.378; P = 0.028) emerged as independent risk factors for the development of SREs during denosumab therapy.The final multivariate logistic regression model demonstrated good fit according to the Hosmer–Lemeshow test (χ^2^ = 4.519, df = 7, P = 0.718). Denosumab treatment duration was retained as a covariate in the multivariate logistic regression model due to its clinical relevance and potential confounding effects, despite not reaching statistical significance in univariate analysis(p = 0.249). Existing evidence suggests an association between treatment duration and bone metastasis progression, and this variable may influence the effect estimates of other predictors. Including this covariate enables more accurate estimation of independent variable effects and enhances the model's clinical applicability and interpretability (Table 2).

**Table 2:** Result of Logistic regression model

Variables		*P*	*OR (*95%*CI)*
Smoking History	No	-	-
	Yes	0.028	3.078(1.131 ~ 8.378)
History of Bisphosphonate Use	No	-	-
	Yes	0.017	0.191(0.049 ~ 0.741)
Corrected pretreatment Serum Calcium (mg/dL)	≥ 8.50	-	-
	< 8.50	0.021	4.913(1.268 ~ 19.044)
Duration of bisphosphonate therapy(months)	< 6		
	6 ~ 9	0.15	0.385 (0.105 ~ 1.413)
	≥ 9	0.502	1.586 (0.413 ~ 6.088)

**Fig. 2:**
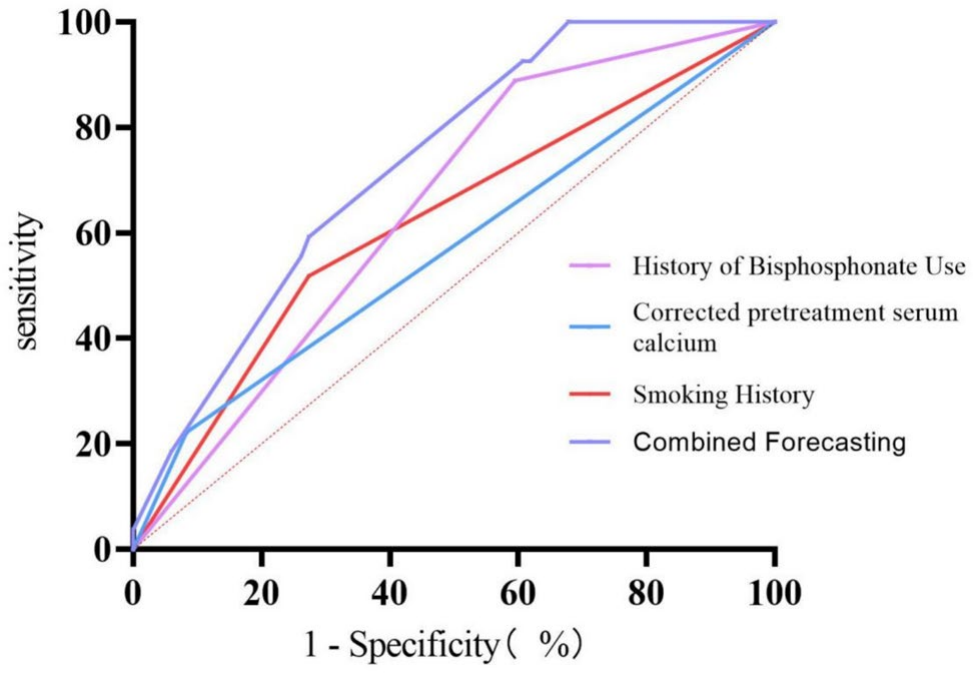
Receiver operating characteristic (ROC) curve for the model of risk factors

Based on the multivariate logistic regression model, individualized predicted probabilities for skeletal-related events (SREs) were calculated for each patient. Receiver operating characteristic (ROC) curve analysis demonstrated that the predictive model had moderate discriminative ability, with an area under the curve (AUC) of 0.731 (95% CI: 0.633–0.829), which was statistically significant (P < 0.001). The optimal predicted probability cutoff value of 8.67% was determined by maximizing the Youden index. At this threshold, the model exhibited a sensitivity of 100.0% and a specificity of 32.1% for identifying patients with SREs (Fig. 2).

## Discussion

Skeletal-related events (SREs) are common and serious complications in patients with stage IV lung adenocarcinoma and bone metastases, substantially impairing quality of life and worsening survival outcomes [13–15]. Although denosumab, a RANKL inhibitor, effectively reduces the incidence of SREs [16, 17], substantial interindividual variability in treatment response exists, and validated predictive models remain lacking. In this study, we conducted multivariable analysis specifically in patients receiving denosumab and suggested prior bisphosphonate use as an independent protective factor (OR, 0.191), while pretreatment hypocalcemia (OR, 4.913) and smoking (OR, 3.078) were independently associated with increased risk of SREs. These findings support the development of risk stratification models and individualized monitoring strategies.

Previous studies have established the efficacy of bisphosphonates in mitigating osteolytic activity through osteoclast inhibition [18], though most prior research has focused on their role as monotherapy for bone metastases [19, 20]. Our study is the first to demonstrate that a history of bisphosphonate use prior to denosumab administration—such as with zoledronic acid—significantly reduces the risk of SREs (OR, 0.191), thereby extending the clinical utility of bisphosphonates. Given that bisphosphonates may influence SRE occurrence by inducing hypocalcemia, we further analyzed their impact on pre-treatment hypocalcemia. The results showed no significant association between prior bisphosphonate use and the incidence of pre-treatment hypocalcemia before denosumab therapy (p = 0.538). This negative finding may be attributed to the relatively short duration of bisphosphonate therapy in our cohort (median 2.6 months), which might be insufficient to exert long-term effects on calcium metabolism. Furthermore, this retrospective analysis has inherent limitations, including incomplete documentation of calcium and vitamin D supplementation. Future prospective studies with systematic long-term data collection on drug metabolism are warranted to validate these findings..Additionally,, emerging evidence suggests that sequential bisphosphonate therapy following prolonged denosumab use may maintain skeletal homeostasis, minimize bone loss, and mitigate the risk of rebound bone turnover upon discontinuation [21, 22]. In light of these findings, clinicians should consider the potential additive value of bisphosphonates when designing bone-targeted strategies for patients with stage IV lung adenocarcinoma and bone metastases, particularly those at high risk for SREs or undergoing treatment transitions.

Our study also identified pretreatment hypocalcemia, defined as a corrected serum calcium level < 8.50 mg/dL, as an independent risk factor for SREs (OR, 3.877). The underlying pathophysiological mechanism may involve disruption of skeletal calcium homeostasis, leading to increased bone fragility and a heightened risk of pathologic fractures and related complications. This may be mediated by elevated bone turnover markers such as C-terminal telopeptide of type I collagen (CTX-1) and suppressed osteocalcin (OC) synthesis [23]. Denosumab itself has been associated with a higher risk of treatment-emergent hypocalcemia [24, 25], which may further predispose patients to SREs. Ulas et al. [10]reported that baseline hypercalcemia was inversely associated with SRE risk (OR, 0.33), supporting the present findings. Additionally, Dennis et al. (2020) [26]showed that baseline vitamin D deficiency (25-OH-vitamin D < 20 ng/mL) significantly impaired intestinal calcium absorption and renal calcium reabsorption via downregulation of calbindin-D9k, increasing the risk of hypocalcemia during bone-modifying therapy (OR, 2.546; P = 0.023). That study also confirmed a protective effect of pre-treatment hypercalcemia (serum calcium > 10.2 mg/dL, OR, 0.474; P = 0.032) against developing hypocalcemia. These mechanistic insights suggest that during denosumab therapy, clinicians should: (1) dynamically monitor serum calcium and 25-OH-vitamin D levels; and (2) implement calcium supplementation strategies in patients with persistent hypocalcemia to optimize skeletal metabolism and reduce the risk of SREs.

Smoking is known to impair bone homeostasis through multiple pathologic mechanisms, including enhanced osteoclast activation and reduced intestinal calcium absorption. Experimental studies have shown that cadmium and nicotine in tobacco smoke can suppress the Wnt/β-catenin signaling pathway, thereby disrupting the coupling of bone formation and resorption [27]. Clinical evidence also links smoking to elevated SRE risk. A Korean cohort study demonstrated that non-small cell lung cancer (NSCLC) patients with a smoking history had a significantly higher SRE incidence than nonsmokers (OR, 2.80; 95% CI, 1.32–6.00) [12]. Our study further establishes that this association holds true for the specific population of patients with bone-metastatic stage IV lung adenocarcinoma undergoing denosumab treatment, in whom smoking was identified as an independent predictor (OR, 3.078; 95% CI, 1.131–8.378; P = 0.028). This may reflect a pharmacodynamic antagonism between tobacco-induced upregulation of RANKL signaling and RANKL inhibition by denosumab. Consequently, smoking status should be incorporated into SRE risk assessment tools, and smoking cessation should be strongly reinforced during antiresorptive therapy.

This study established a predictive model for assessing skeletal-related event (SREs) risk in patients with bone-metastatic lung adenocarcinoma by incorporating smoking history, pretreatment hypocalcemia, and prior bisphosphonate use. The model demonstrated good discriminative capacity, with an area under the curve (AUC) of 0.731. The optimal decision threshold was determined as 8.67% using the Youden index. At this cutoff value, the model achieved 100% sensitivity while maintaining limited specificity (32.1%), ensuring comprehensive identification of high-risk patients. This quantitative tool provides an objective basis for clinical risk stratification, facilitates intensified monitoring of high-risk individuals and optimized allocation of medical resources, thereby offering practical evidence for precision management in supportive cancer care.

Several limitations of this study merit consideration. First, as a single-center retrospective analysis with a relatively modest sample size (n = 111), our findings may be subject to selection and information biases. Second, although prior bisphosphonate use emerged as a strong predictor, we did not stratify by dose, duration, or transition regimens (e.g., zoledronic acid 4 mg vs. 8 mg), which may limit the generalizability of our findings. Future studies should adopt multicenter prospective cohort designs with larger sample sizes, incorporate pharmacokinetic variables (e.g., denosumab dosing intervals), and include genomic data (e.g., RANKL expression levels or vitamin D receptor polymorphisms),to improve model specificity while maintaining high sensitivity, thereby establishing a more clinically applicable risk prediction tool.

## Conclusion

In this retrospective study of patients with stage IV non–small-cell lung cancer and bone metastases treated with denosumab, prior bisphosphonate use was identified as an independent protective factor against skeletal-related events (OR, 0.191), while pretreatment hypocalcemia (OR, 4.913) and smoking (OR, 3.078) were independently associated with elevated risk. These findings underscore the importance of risk stratification and enhanced surveillance in vulnerable patient subgroups.

## Data Availability

The datasets used or analysed during the current study are available from the corresponding author on reasonable request.

## References

[1] Bray F, Laversanne M, Sung H, Ferlay J, Siegel RL, Soerjomataram I, Jemal A (2024) Global cancer statistics 2022: GLOBOCAN estimates of incidence and mortality worldwide for 36 cancers in 185 countries. CA Cancer J Clin 74(3):229–263. 10.3322/caac.21834 38572751 10.3322/caac.21834

[2] Nicholson AG, Tsao MS, Beasley MB, Borczuk AC, Brambilla E, Cooper WA, Dacic S, Jain D, Kerr KM, Lantuejoul S, Noguchi M, Papotti M, Rekhtman N, Scagliotti G, van Schil P, Sholl L, Yatabe Y, Yoshida A, Travis WD (2022) The 2021 WHO classification of lung tumors: impact of advances since 2015. J Thorac Oncol 17(3):362–387. 10.1016/j.jtho.2021.11.003 34808341 10.1016/j.jtho.2021.11.003

[3] Hong S, Youk T, Lee SJ, Kim KM, Vajdic CM (2020) Bone metastasis and skeletal-related events in patients with solid cancer: a Korean nationwide health insurance database study. PLoS ONE 15(7):e0234927. 10.1371/journal.pone.0234927 32678818 10.1371/journal.pone.0234927 PMC7367479

[4] Zhou Y, Yu QF, Peng AF, Tong WL, Liu JM, Liu ZL (2017) The risk factors of bone metastases in patients with lung cancer. Sci Rep 7(1):8970. 10.1038/s41598-017-09650-y 28827719 10.1038/s41598-017-09650-y PMC5567132

[5] Kong P, Yan J, Liu D, Ji Y, Wang Y, Zhuang J, Wang J, Hu X, Yue X (2017) Skeletal-related events and overall survival of patients with bone metastasis from nonsmall cell lung cancer - a retrospective analysis. Medicine (Baltimore) 96(51):e9327. 10.1097/md.0000000000009327 29390509 10.1097/MD.0000000000009327 PMC5758211

[6] Shimada H, Setoguchi T, Yokouchi M, Sasaki H, Ishidou Y, Kawamura I, Abematsu M, Nagano S, Komiya S (2014) Metastatic bone tumors: analysis of factors affecting prognosis and efficacy of CT and (18)F-FDG PET-CT in identifying primary lesions. Mol Clin Oncol 2(5):875–881. 10.3892/mco.2014.326 25054061 10.3892/mco.2014.326 PMC4106751

[7] LeVasseur N, Clemons M, Hutton B, Shorr R, Jacobs C (2016) Bone-targeted therapy use in patients with bone metastases from lung cancer: a systematic review of randomized controlled trials. Cancer Treat Rev 50:183–193. 10.1016/j.ctrv.2016.09.013 27716496 10.1016/j.ctrv.2016.09.013

[8] De Castro J, García R, Garrido P, Isla D, Massuti B, Blanca B, Vázquez J (2015) Therapeutic potential of denosumab in patients with lung cancer: beyond prevention of skeletal complications. Clin Lung Cancer 16(6):431–446. 10.1016/j.cllc.2015.06.004 26264596 10.1016/j.cllc.2015.06.004

[9] Lipton A, Fizazi K, Stopeck AT, Henry DH, Smith MR, Shore N, Martin M, Vadhan-Raj S, Brown JE, Richardson GE, Saad F, Yardley DA, Zhou K, Balakumaran A, Braun A (2016) Effect of denosumab versus zoledronic acid in preventing skeletal-related events in patients with bone metastases by baseline characteristics. Eur J Cancer 53:75–83. 10.1016/j.ejca.2015.09.011 26693901 10.1016/j.ejca.2015.09.011

[10] Ulas A, Bilici A, Durnali A, Tokluoglu S, Akinci S, Silay K, Oksuzoglu B, Alkis N (2016) Risk factors for skeletal-related events (SREs) and factors affecting SRE-free survival for nonsmall cell lung cancer patients with bone metastases. Tumour Biol 37(1):1131–1140. 10.1007/s13277-015-3907-z 26276360 10.1007/s13277-015-3907-z

[11] Katakami N, Kunikane H, Takeda K, Takayama K, Sawa T, Saito H, Harada M, Yokota S, Ando K, Saito Y, Yokota I, Ohashi Y, Eguchi K (2014) Prospective study on the incidence of bone metastasis (BM) and skeletal-related events (SREs) in patients (pts) with stage IIIB and IV lung cancer-CSP-HOR 13. J Thorac Oncol 9(2):231–238. 10.1097/jto.0000000000000051 24419421 10.1097/JTO.0000000000000051 PMC4132043

[12] Sun JM, Ahn JS, Lee S, Kim JA, Lee J, Park YH, Park HC, Ahn MJ, Ahn YC, Park K (2011) Predictors of skeletal-related events in non-small cell lung cancer patients with bone metastases. Lung Cancer 71(1):89–93. 10.1016/j.lungcan.2010.04.003 20598769 10.1016/j.lungcan.2010.04.003

[13] Song Q, Shang J, Zhang C, Zhang L, Wu X (2019) Impact of the homogeneous and heterogeneous risk factors on the incidence and survival outcome of bone metastasis in NSCLC patients. J Cancer Res Clin Oncol 145(3):737–746. 10.1007/s00432-018-02826-7 30603904 10.1007/s00432-018-02826-7 PMC11810367

[14] Zhang H, Zhu W, Biskup E, Yang W, Yang Z, Wang H, Qiu X, Zhang C, Hu G, Hu G (2018) Incidence, risk factors and prognostic characteristics of bone metastases and skeletal-related events (SREs) in breast cancer patients: a systematic review of the real world data. J Bone Oncol 11:38–50. 10.1016/j.jbo.2018.01.004 29511626 10.1016/j.jbo.2018.01.004 PMC5832676

[15] Santini D, Barni S, Intagliata S, Falcone A, Ferraù F, Galetta D, Moscetti L, La Verde N, Ibrahim T, Petrelli F, Vasile E, Ginocchi L, Ottaviani D, Longo F, Ortega C, Russo A, Badalamenti G, Collovà E, Lanzetta G, Mansueto G, Adamo V, De Marinis F, Satolli MA, Cantile F, Mancuso A, Tanca FM, Addeo R, Russano M, Sterpi M, Pantano F, Vincenzi B, Tonini G (2015) Natural history of non-small-cell lung cancer with bone metastases. Sci Rep 5:18670. 10.1038/srep18670 26690845 10.1038/srep18670 PMC4687045

[16] Martin M, Bell R, Bourgeois H, Brufsky A, Diel I, Eniu A, Fallowfield L, Fujiwara Y, Jassem J, Paterson AH, Ritchie D, Steger GG, Stopeck A, Vogel C, Fan M, Jiang Q, Chung K, Dansey R, Braun A (2012) Bone-related complications and quality of life in advanced breast cancer: results from a randomized phase III trial of denosumab versus zoledronic acid. Clin Cancer Res 18(17):4841–4849. 10.1158/1078-0432.Ccr-11-3310 22893628 10.1158/1078-0432.CCR-11-3310

[17] Wang Z, Qiao D, Lu Y, Curtis D, Wen X, Yao Y, Zhao H (2015) Systematic literature review and network meta-analysis comparing bone-targeted agents for the prevention of skeletal-related events in cancer patients with bone metastasis. Oncologist 20(4):440–449. 10.1634/theoncologist.2014-0328 25732263 10.1634/theoncologist.2014-0328 PMC4391764

[18] Wong MH, Stockler MR, Pavlakis N (2012) Bisphosphonates and other bone agents for breast cancer. Cochrane Database Syst Rev 2:1003474. 10.1002/14651858.CD003474.pub3 10.1002/14651858.CD003474.pub3 22336790

[19] Battafarano G, Rossi M, Marampon F, Del Fattore A (2018) Cellular and molecular mediators of bone metastatic lesions. Int J Mol Sci. 10.3390/ijms19061709 29890702 10.3390/ijms19061709 PMC6032429

[20] Jara MA, Varghese J, Hu MI (2022) Adverse events associated with bone-directed therapies in patients with cancer. Bone 158:115901. 10.1016/j.bone.2021.115901 33631354 10.1016/j.bone.2021.115901

[21] McDonough A, Malomo K, Brennan F, Fallon N, Steen G, Maher N, O’Carroll C, Walsh JB, Lannon R, McCarroll K (2022) Treatment challenges when stopping denosumab. Ir Med J 115(3):567 35532944

[22] Lamy O, Stoll D, Aubry-Rozier B, Rodriguez EG (2019) Stopping denosumab. Curr Osteoporos Rep 17(1):8–15. 10.1007/s11914-019-00502-4 30659428 10.1007/s11914-019-00502-4

[23] Bager CL, Bay F, Christiansen C, Karsdal M (2019) Low bone turnover levels predict increased risk of cancer. Bone 127:75–81. 10.1016/j.bone.2019.05.032 31150870 10.1016/j.bone.2019.05.032

[24] Ikesue H, Tsuji T, Hata K, Watanabe H, Mishima K, Uchida M, Egashira N, Miyamoto T, Baba E, Akashi K, Takayama K, Nakanishi Y, Tokunaga E, Okamoto T, Maehara Y, Yokomizo A, Naito S, Kubo M, Tanaka M, Masuda S (2014) Time course of calcium concentrations and risk factors for hypocalcemia in patients receiving denosumab for the treatment of bone metastases from cancer. Ann Pharmacother 48(9):1159–1165. 10.1177/1060028014539919 24928100 10.1177/1060028014539919

[25] Body JJ, Bone HG, de Boer RH, Stopeck A, Van Poznak C, Damião R, Fizazi K, Henry DH, Ibrahim T, Lipton A, Saad F, Shore N, Takano T, Shaywitz AJ, Wang H, Bracco OL, Braun A, Kostenuik PJ (2015) Hypocalcaemia in patients with metastatic bone disease treated with denosumab. Eur J Cancer 51(13):1812–1821. 10.1016/j.ejca.2015.05.016 26093811 10.1016/j.ejca.2015.05.016

[26] White PS, Dennis M, Jones EA, Weinberg JM, Sarosiek S (2020) Risk factors for development of hypocalcemia in patients with cancer treated with bone-modifying agents. J Natl Compr Canc Netw 18(4):420–427. 10.6004/jnccn.2019.7370 32259788 10.6004/jnccn.2019.7370

[27] Need AG, Kemp A, Giles N, Morris HA, Horowitz M, Nordin BE (2002) Relationships between intestinal calcium absorption, serum vitamin D metabolites and smoking in postmenopausal women. Osteoporos Int 13(1):83–88. 10.1007/s198-002-8342-9 11883410 10.1007/s198-002-8342-9

